# Annotations

**Published:** 1895-04-20

**Authors:** 


### Annotations.

### Does Novel-Reading Prevent Marriage?

A writer in a monthly review, discussing Mr. Balfour's "Foundations of Belief," takes occasion to say that solid books, dealing with the great problems of mind and morals, are no longer read, except by a few specialists. That an exclusive diet of novel-reading is extremely debilitating is proved by one series of facts which are observable in every part of the civilised world. Men and women among the reading classes no longer marry in anything like such numbers as they formerly did, and the reason is that they have no pluck in them to face lives of Spartan simplicity on limited incomes. The result is disastrous to women, inasmuch as it prevents many of them ever marrying at all. For if a woman does not marry when she is young very few men care to marry her when she is middle-aged. Men marry in middle life, but they do not marry women of their own age; they marry young women. The physiological moral is that it would be vastly better for both men and women to read novels for recreation only; and when at work to read solid books which really exercise and develop the brain. In practice the result of this would be that both men and women would have better and stronger brains. They would marry earlier and with more courage; they would face the world more hopefully and successfully, and they would become the parents of wholesomer, healthier, happier, and more capable children.

### "One Step" at the Tottenham Hospital.

The dispute at the Tottenham Hospital between the medical staff on the one hand, and a portion of the nursing staff with some of the managers on the other, has been advanced by a significant step. The members of the medical profession practising at Tottenham have taken up the question, and by a vote of practically the whole of their body have expressed their full confidence in, and their entire sympathy with, Dr. Hooper May, the senior surgeon, and his colleagues, and also their conviction that a competent investigation will result in peace and improved management. Moreover, we happen to know at first hand that certain medical men of consultant status in London who have been approached with a view to appointment, have expressed their sympathy with the medical staff, and their determination to accept no appointments until justice is done and peace is restored. The decided measures of the local practitioners and the London consultants have a very decided significance. They leave the responsible managers no alternative but that of arranging for a competent investigation by a judicial tribunal, composed mainly of outsiders and experts, whose decisions will be above suspicion and absolutely final. There can be no possible objection to such an investigation. Nobody, so far as we know, has anything serious to conceal. We do not imagine that the most microscopic investigation will bring any scandals to light. There has simply been a little too much zeal in one direction, which has led to differences of opinion and misunderstandings, together with a condition of friction which makes it impossible for the hospital to do its work in the spirit and with the efficiency which ought always to characterise hospital work. Reason, we hope, will now prevail; investigation will show how small, after all, the causes of quarrel have been; peace will be restored, and the hospital will do its good work again, just as it did before the misunderstandings arose.

### Weighing the Baby.

There are few more curious prejudices than that against weighing a new-born infant. It is held to bring ill-luck, and both mothers and midwives will be found in many cases to protest against any such thing being done. The origin of the superstition is by no means easy to discover; probably it is but one expression of the far too common idea that the luck of infants, their good or their ill health, is in some inscrutable way Divinely ordered, and that any interference is but tempting Providence. Against all such negation of responsibility we must protest. Dame Nature is a good creature, and may be trusted to do right, but bottle-fed babies are not Dame Nature's handiwork. So long as women fulfilled their maternal duties, the necessity for anxious inquiry as to whether the infant's food was being digested was not so urgent; but when, instead of nursing their babies they elect to bring them up by hand, and to feed them on various curious compounds which may or may not agree, every hint as to whether or not the food so given is being absorbed becomes important. Weighing the baby, then, should never be neglected; for however well and happy it may seem, if it is not putting on its normal addition of weight there is something wrong. In adults, loss of weight is one of the most important signs of diseases which interfere with nutrition; but in infants it is not a question merely of loss of weight, any lessening of the normal gain should attract attention, for long before the various troubles of digestion to which hand-fed infants are so liable show themselves by general symptoms, or by wasting, they will interfere with that steady increase of weight which week by week a healthy infant shows. Dr. Griffith, lecturing on infantile disorders, points out that for from three to five days after birth it is common for children to lose weight. During that time they lose meconium and urine, and water is evaporated in respiration, while they have not yet begun to absorb their full supply of nutriment. At the end of the first week, however, the baby should have made up its weight to what it was at birth. Then from the end of the first week it should gain an ounce a day. "A baby," he says, "which gains half-an-ounce a day is doing fairly well, but a child doing thoroughly well gains double that amount or more. This should continue until the fourth month, after which a daily gain of half-an-ounce a day is satisfactory." Accurate observation and careful recording of the weight of an infant gives the earliest warning of digestive troubles, and should be looked on in the light of a duty by those who undertake the responsible task of bringing up a child by hand, for on the success with which the child digests the food presented to it depends in large degree the perfection of the framework on which the future man is built.

